# HIV Education, Empathy, and Empowerment (HIVE^3^): A Peer Support Intervention for Reducing Intersectional Stigma as a Barrier to HIV Testing among Men Who Have Sex with Men in Ghana

**DOI:** 10.3390/ijerph182413103

**Published:** 2021-12-12

**Authors:** Gamji M’Rabiu Abubakari, Francis Owusu-Dampare, Adedotun Ogunbajo, Joseph Gyasi, Michael Adu, Patrick Appiah, Kwasi Torpey, Laura Nyblade, LaRon E. Nelson

**Affiliations:** 1 Center for Interdisciplinary Research on AIDS, School of Public Health, Yale University, New Haven, CT 06510, USA; laron.nelson@yale.edu; 2 Priorities on Rights and Sexual Health, Accra 00233, Ghana; fodd007@gmail.com; 3 Department of Epidemiology, Harvard T.H. Chan School of Public Health, Boston, MA 02115, USA; adedotun_ogunbajo@alumni.brown.edu; 4 School of Nursing, Yale University, New Haven, CT 06477, USA; joseph.gyasi@yale.edu; 5 Youth Alliance for Health & Human Rights, Kumasi 00233, Ghana; adumiky@gmail.com (M.A.); ryanappiah@yahoo.com (P.A.); 6 School of Public Health, University of Ghana, Accra 00233, Ghana; ktorpey@ug.edu.gh; 7 Global Health Division, International Development Group Research Triangle Institute International, New York, NY 10001, USA; lnyblade@rti.org; 8 St Michael’s Hospital Centre for Urban Health Solutions, Toronto, ON M5B 1W8, Canada

**Keywords:** HIV testing, peer communication support, HIV testing communication, men who have sex with men, community-based research, mobile-based intervention, Africa, ADAPT-ITT model, dennis peer support model, intersectional stigma

## Abstract

Men who have sex with men (MSM) in Ghana remain at heightened risk of HIV infection, and face challenges in accessing HIV prevention and care services. Previous research in Ghana shows that MSM face intersectional stigma across ecological levels (family, peers, healthcare settings, and community level) and the criminalization of same-gender sexual behaviors in the country. To protect their wellbeing from exposure to stigma, many MSM avoid interactions with healthcare systems and services, which inadvertently inhibits their opportunities for early detection and treatment of HIV. Consequently, MSM in Ghana carry a disproportionate burden of HIV prevalence (18%) compared to the general population (2%), highlighting the need for culturally relevant processes in HIV/STI prevention, and care communication to optimize sexual health and wellness among MSM in Ghana. To this effect, we collaborated with community partners to use the Assessment, Decision, Adaptation, Production, Topical Experts, Training, Testing (ADAPT-ITT) model to modify a theory-driven smartphone-based peer support intervention to enhance its focus on intersectional stigma reduction, and improve HIV health-seeking behaviors among MSM, including HIV testing and linkage to care. We used the Dennis Peer Support Model to develop the peer support components (emotional, informational, and appraisal support) to increase peer social support, decrease social isolation, and minimize intersectional stigma effects on HIV-related healthcare-seeking behaviors. This paper shows the preliminary acceptability and effectiveness of employing culturally relevant techniques and communication strategies to provide secure peer support to improve HIV prevention and care among key populations in highly stigmatized environments.

## 1. Introduction

In Ghana, men who have sex with men (MSM) experience multilevel stigma due to intersecting marginalized identities (e.g., sexual orientation, gender identity, HIV status) that contribute to adverse health outcomes [1,2]. In Ghana, same-gender sexual behavior may be punishable by up to three years of imprisonment [3]. A new bill submitted to the Ghanaian parliament in June 2021 would make identifying as gay or an ally to the LGBTQ+ community a second-degree felony, punishable by up to five years imprisonment. Advocating for the human rights of LGBTQ-identified Ghanaians would be punishable by up to 10 years imprisonment. Together with societal homophobia and anti-LGBTQ attitudes, these state-sanctioned discriminatory policies facilitate an environment where MSM constantly experience stigma, discrimination, violence, and prejudice [2,4,5].

MSM in Ghana remain disproportionately affected by the HIV epidemic, with an estimated prevalence of 18% compared to 2% for the general population [6,7,8]. Factors contributing to the increased risk of HIV transmission among MSM in Ghana include low rates of HIV testing, and stigma associated with HIV testing, insufficient knowledge of HIV pre-exposure prophylaxis (PrEP), and inconsistent condom use [2,9,10,11,12]. Additionally, MSM living with HIV in Ghana experience various barriers to receiving quality healthcare services, such as experiences of stigma, unaffordability of healthcare services, long wait times at medical facilities, and poor patient–provider relationships [9,10,11,12].

Interventions that incorporate peer communication, support, and social networking strategy (SNS) to facilitate linkage to HIV prevention and care services for MSM might help increase uptake and adherence for this marginalized community [13,14,15]. A pilot study to assess the feasibility of using social network strategy to identify and refer MSM to HIV testing and counseling services was shown to be highly effective in recruiting MSM who never tested for HIV, and were unaware of their HIV serostatus, and identified new cases of HIV [16]. A recently published lesson on three community-based HIV interventions with MSM in Ghana recommends incorporating elements of effective communication in peer support through peer mentoring and mobile applications to facilitate communication [13]. This provides preliminary evidence for incorporating peer-to-peer communication and support, and SNS in HIV prevention and treatment approaches for stigmatized populations, such as MSM in Ghana.

For the current study, we employed a health promotion approach to collaborate with community partners in using the ADAPT-ITT model to adapt HIV Education, Empathy, and Empowerment (HIVE^3^) Intervention to train MSM in Ghana to facilitate effective sexual health communication through peer mentoring and support. We specifically aimed to use peer support to address intersectional stigma, and improve HIV health-seeking behaviors among MSM, including HIV testing, and linkage to care using a mobile platform and effective communication strategies [1]. We used the Dennis Peer Support Model to develop the peer support components (emotional, informational, and appraisal support) to increase peer social support, decrease social isolation, and minimize intersectional stigma effects on HIV-related healthcare-seeking behaviors [17,18].

## 2. The Dennis Peer Support Model

Peer support reflects the giving of assistance and encouragement by an individual considered equal [17,18]. Peer support has been successfully provided through multiple modes of interactions, such as individual one-on-one sessions, or self-help support groups, and in diverse settings, such as homes, hospitals, and schools [17,18] The model assumes that peers act as catalysts for change, and understand the target population’s unique situations hidden from other embedded social networks. Influencing behavioral change relies heavily on trust; peers can command such confidence, inspire and facilitate the needed change. Three attributes serve as the supportive function of peer relationships and apply to various stressors and health outcomes in multiple ways. In this study, we train peers to provide support around these three attributes: emotional; informational; and appraisal/affirmation support. *Emotional Support*—emotionally supportive interactions include expressions of caring, reassurance, attentive listening, reflections, and encouragement to people facing challenges with coping. Such exchanges can help the affected person feel cared for, accepted, admired, and valued when successfully implemented [18,19,20]. *Informational Support*—the provision of knowledge relevant to problem-solving, including the availability of appropriate resources, independent assessments, alternative courses of action, and guidance about effectiveness [20,21,22]. *Appraisal or Affirmational Support*—the communication of pertinent information to support self-evaluation and expressions that affirm the appropriateness of emotions, cognitions, and behaviors. When people experience setbacks, providing a safe environment for them to express themselves freely, while conveying thoughts that encourage persistence in problem resolution, assistance to endure frustration, and the communication of optimism, helps them find the motivation they need to bounce back [22,23,24].

In this paper, we describe the intervention and the adaptation process to serve as a guide for scholars and program implementers with interest in adapting interventions that will employ culturally relevant techniques and communication strategies to provide secured peer support targeted at improving HIV prevention and care among key populations in highly stigmatized environments. Our team created the currently adapted intervention to link people living with HIV with their peers, who can relate to their experiences living with HIV, and support them via effective information sharing, emotional connection, and linkage to nurses via a mobile platform [1].

## 3. The Approach Employed in Adapting HIVE^3^

These steps described reflect the guidelines provided by the ADAPT-ITT framework to guide intervention adaptation. We explain the detailed steps in our previous article [9]. The model consists of eight components that drive the adoption or adaptation of evidence-based interventions to fit the cultural context of the population of interest [9,24–27]. The steps include: (1) *Assessment*—establishing the need of intervention; (2) *Decision*—deciding on the intervention to be adapted; (3*) Adaptation*—theatre testing with the target population, and identifying changes to be implemented; (4) *Production*—producing draft manual contents; (5) *Topical Experts*—soliciting expert review of contents, and recommendations for improvement; (6) *Integration*—analyzing and incorporating feedback; (7) *Training*—building capacity of intervention implementation team; (8) *Testing—*pilot testing of the adapted intervention [9,24–27].

### 3.1. ADAPT-IT Step One—Assessment

*Preliminary Data Collection.* We conducted qualitative interviews (*n* = 10) with MSM living with HIV and focus groups (*n* = 8) among MSM irrespective of their HIV status. The interviews and focus groups seek to understand the circumstances or experiences surrounding diagnosis, specifically the intersection of HIV, gender nonconformity, and same-sex stigmas within the community and health care settings. The interviews also seek to understand the experiences of MSM living with HIV with medical care after diagnosis, specifically, provider interactions, and barriers to HIV care engagement.

*Sampling and Recruitment**.*** The participants reside in Ghana’s two largest cities, Accra and Kumasi, with the highest HIV prevalence rates among MSM (Accra, 42%; Kumasi, 25%). We trained two partner MSM community-based organizations on qualitative interviewing and ethics. They recruited the MSM from client networks, and distributed coupons to reach others via snowballing. The sample for focus groups included 18-years or older cis-gender men who reported having same-sex intercourse within the previous six months. For interviews, MSM had to meet the focus group criteria, and self-disclose living with HIV. Before the interviews, we received ethical approval from the Ghana Health Service, Yale University, Noguchi Memorial Institute for Medical Research, and the University of Toronto IRBs.

**Figure 1:**
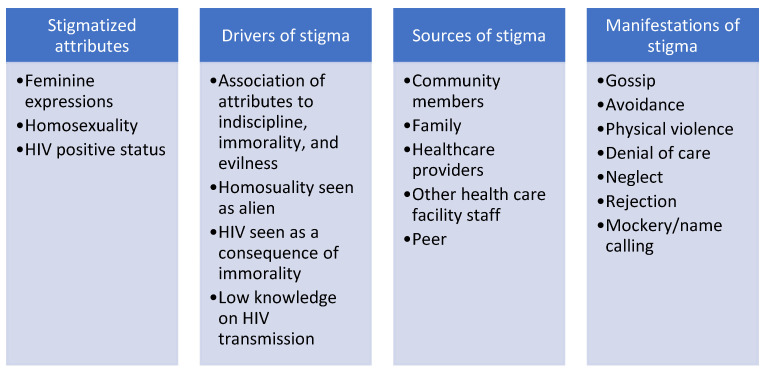
Snapshot of Rapid Analaysis Results.

*Analysis and results**:*** We transcribed the audio files of the qualitative data. We conducted a rapid analysis of the data by reviewing and creating a single summative report of the key findings to inform the adaptation of the intervention. Four team members reviewed the transcripts and summarized the contents of the transcripts individually. Two team members compiled the summaries into a single report. *Snapshot of results*: As shown in Figure 1, participants reported facing intersectional stigma at multiple levels (e.g., family, community members, friends, and healthcare workers), and expressed interest in working with MSM-friendly organizations compared to general hospitals. Participants identified the normalized linkage of same-gender sexual activities with foreign culture, immorality, evil, and indiscipline as driving factors of their experiences of intersectional stigma in Ghana. We also observed internalized same-gender stigma, gender nonconformity stigma, and HIV stigma among some participants. They recommended that interventions address HIV risk and stigma among MSM and health care settings. Detailed qualitative processes and findings are reported elsewhere.

### 3.2. ADAPT-IT Steps Two—Decision, Three—Adaptation, and Four—Production

Based on the findings of the assessment step, we deemed our initially designed intervention HIVE^3^ fit to address MSM internalized, peer-to-peer intersectional stigma and to buffer effects of experienced intersectional stigma in health care facilities. All experiences of intersectional stigma associates with same-gender sex, gender nonconformity, and HIV. The intervention also fits with recommendations on addressing HIV risk and increasing access to HIV testing among MSM in the country. We formed a team led by the first author, with members including Yale research assistant, JG, and community partners, FOD and MA, to review the intervention. At this stage, we did not pilot test the intervention as it was previously tested among MSM in Ghana [1]. However, as shown in subsequent sections, we highlighted the critical components of the intervention, and identified areas to submit to experts for review in the next step of the adaptation process.

### 3.3. ADAPT-IT Steps Five—Topical Experts and Six—Integration

We submitted the summative report and the HIVE^3^ manual to experts in HIV science, and gender and sexuality, community-level stakeholders, nurses, and critical MSM community organization leaders in Ghana. Experts provided written recommendations, and contributed to a workshop discussion over Zoom on ways to adapt the intervention to meet the needs of MSM based on the data and their experience working with MSM in Ghana and sub-Saharan African countries.

*Recommendations*. The previous HIVE^3^ mainly focused on HIV information and services. Hence, experts recommended emphasizing intersectional stigma, especially through interactive and reflective activities, addressing confidentiality among peer mentors, and effective communication strategies, especially regarding personal level issues, such as status, outing, and disclosure. Moreover, they recommended psyching the mentors to respond to non-HIV-related concerns, such as community-level stigma, human rights, and basic needs crisis questions. Researchers and MSM community partners (*n* = 4), led by GMRA, reviewed the previous manual, and created a new manual that incorporated exercises to address the adaptation recommendations. We sent the new manual draft to the creator of the peer support model used for the intervention to review it for its fidelity to the model [17,18]. We received a confirmation of its fidelity, and suggestions on organizations, and clarifications on delivering the contents of the theory. We then moved the manual to final production.

**Step by step description of the current HIVE^3^.** The manual consists of six sessions which we implemented chronologically (Table 1). Each session includes a description of objectives, specific sub-modules, an explanation of critical activities, a step-by-step instructor note (guidelines), and a description of materials needed in training the peer mentors. As seen in Table 1, sessions 1–6, the session breakdown includes the following: (1) Building Rapport and Confidentiality between Peer Mentors, Mentees, and other Team Members; (2) Understanding Intersectional Stigma and How it Impacts HIV Care for MSM; (3) The Dennis Peer Support Model and the Role of Peer Mentors; (4) Effective Communication in Delivering Peer Support; (5) Self-Efficacy; (6) Other Areas of Support, Usage of HIVE^3^ Platform, Signing Up.

*(1) Building Rapport and Confidentiality between Peer Mentors, Mentees, and other Team Members.* Session one focuses on building rapport between facilitators and mentors by creating space for familiarization, and setting a conducive environment for the training. It has four modules labeled A to E. In A, facilitators lead mentors to build rapport by creating interactive fun activities and introductory sessions. They transition to B, where they set the ground rules. In C, they discuss critical expectations of the workshop, general study outlook, and objectives, emphasizing the purpose of the entire mentoring intervention. The group then moves to discuss specifics of some of the characteristics of a good mentor in C. Afterwards, they discuss confidentiality through a thought-provoking exercise that allows them to self-reflect on the importance of confidentiality, and the consequences of breaking confidentiality for both the mentor and the peer. By completing the first session, mentors boost their confidentiality levels, appreciate the need for confidentiality, and create a safe space for interactions for the remainder of the training.

*(2) Understanding Intersectional Stigma and How it Impacts HIV Care for MSM.* This session has three modules (A–C) that focus on leading mentors to examine intersectional stigma within the Ghanaian community, MSM community, and health care settings. A introduces mentors to the concepts of stigma and discrimination, and the multiple layers of stigma that could interact to impact MSM. In B, mentors hold an open dialogue forum, and reflect, share, and discuss their personal experiences of intersectional stigma as MSM, and brainstorm the impacts of intersectional stigma on MSM living with HIV in C. By completing session 2, mentors would appreciate the connection between stigma and health-seeking behaviors/experiences (e.g., HIV testing and retention to care), and ways to reduce intersectional stigma.

*(3) The Dennis Peer Support Model and the Role of Peer Mentors.* This session uses six modules (A-F) to examine the Dennis Peer Support Model (DPSM) and its components (emotional, informational, and appraisal). Additionally, the session examines the role of peer mentors in providing support on dealing with intersectional stigma, receiving MSM, and supporting MSM with self-efficacy development. A introduces mentors to the DPSM model and the various components as it applies to HIVE^3^. B examines emotional support in detail in relation to intersectional stigma and HIV testing and identifies how to provide emotional support using life experiences. In C, mentors examine and establish a detailed understanding of informational support, relate it to intersectional stigma and HIV testing, and identify how to provide informational support using life experiences. In D, mentors examine appraisal/affirmational support in detail, and relate it to intersectional stigma and HIV testing, diagnoses, and linkage to care scenarios to identify how to provide emotional support using life experiences. In E, peer mentors attempt to identify and differentiate between the three components of peer support. They then reflect and identify role-plays that will increase mentors’ understanding of practical instances where their support may be needed, and the kind of support they would need to provide in E.

*(4) Effective Communication in Delivering Peer Support.* This session (modules A–C) examines how peer mentors can implement emotional and appraisal/affirmatory support using strategies for effective communications, empathetic listening, texting, and the strategy of LARA—Listen, Affirm, Respond, and Add. A exposes mentors to various communication strategies that could enhance their quality of communication as mentors. B exposes mentors to how to develop empathetic listening and texting when delivering mentorship. C seeks to reinforce the earlier lessons on listening and texting by showing mentors how to communicate effectively and in a non-violent manner.

*(5) Self-Efficacy.* This session examines ways to develop self-efficacy and effective communication on sexual health, HIV, and risk reduction strategies among MSM. Module A aims to increase understanding of self-efficacy and ways to develop self-efficacy. B focuses on tools used in developing self-efficacy. C focuses on understanding how self-talk affects self-efficacy. D helps mentors understand and develop interview skills, which they practice in E. F focuses on developing self-care and effective coping skills among mentors.

*(6) Other Areas of Support, Usage of HIVE^3^ Platform, Signing Up.* This session aims to build the capacity of peer mentors in dealing with issues beyond HIV, gender, and sexual health-related issues that affect MSM. Though it is not part of the scope of their work, it remains essential for them to be aware of how to respond if mentees bring up such issues in their discussion. The session will develop the knowledge and skills to deal with other problems, such as economic stress and different types of stress that MSM go through. This session will also train peer mentors to use the mobile application to chat with peer groups from the HIVE^3^ platform. A summarizes previous sessions, and provides an opportunity to address the essential questions. B discusses economic stress and how to deal with it when mentees ask. C seeks to train mentors on how to use the mobile application to communicate with mentees. 

### 3.4. ADAPT-IT Steps Seven—Training and Eight—Pilot Testing

We organized a two-day retreat-style workshop in Kumasi for our selected peer mentors (*n* = 8). We plan that each mentor will have between 8 to 13 mentees over six months. We randomized 240 participants into intervention (*n* = 122) and control groups. Some (*n* = 19) people did not participate due to fear of insecurity concerning recent homophobia, and the anti-LGBTQ bill and its discourse in public, or because of conflicts with scheduling or other personal concerns.

*“I am afraid that I might get into trouble. Looking at the current situation in the country on LGBTQI+ communities, I don’t feel comfortable even though you have given me all the assurance on security. Thank you for the opportunity, but I can’t continue with the study”— A participant explaining his reasons for dropping out of the study.*

Participants received phones and periodic internet bundles. They communicated via WhatsApp and text. Previously, an app was used, but the availability of WhatsApp, and its familiarity among participants and mentors, made it a more accessible platform. However, we provided them with new numbers, and emphasized the need to remain anonymous in their interactions, and use the phones only for HIVE^3^ activities. We also discouraged them from exchanging personal contacts.

**Preliminary findings.** Participants (*n* = 50) contacted mentors an average of 3 to 4 times for various forms of support within a month of implementation. About 14 inactive participants had challenges with phone cards, and the community partners are currently working on replacing such cards. A rapid review of the support shows that they primarily cluster around information support and emotional support. They mainly ask for information on STI and HIV treatment, current anti-LGBTQ law, and security tips within a highly volatile situation. They request information on understanding gender and sexuality, PrEP, HIV testing, and how to convince their partners to test. Many ask for ways to cope, and share their struggle with their HIV status, and fears in their communities. Detailed analysis will be conducted and published upon completion of the intervention.

## 4. Discussion of Lessons Learned

This paper describes an adapted HIVE^3^, a peer-to-peer support platform that facilitates increased communication between MSM in Ghana on sexual health needs. Like previous interventions, we used the ADAPT-ITT model to adapt the original HIVE^3^ into its current form to address the support needs of a general MSM population, instead of MSM living with HIV as initially implemented [9,25,26,27]. Whereas we maintained the focus of the peer support model that informed the development of the intervention, we made modifications to include current health concerns, such as intersectional stigma and identity among MSM in Ghana. Our experience in adapting and implementing the current intervention reveals lessons that can inform the adaptation of interventions to address the health communication needs of key populations, such as MSM.

Our success in adapting the intervention can largely be attributed to our adherence to the guidelines provided by the ADAPT-ITT model for adopting or adapting interventions. As shown in our previous work, the ADAPT-ITT model has proven effective in adapting interventions across various behavioral health concerns and target populations. Various scholars have used it to adapt HIV interventions. However, some have also applied the framework in implementing other forms of interventions, such as substance use [9,25,26,27].

Peer-to-peer communication interventions can buffer the effects of external threats in addressing HIV prevention and testing among MSM and key populations in highly stigmatized environments. In Ghana, we initiated the study before the introduction of the new anti-LGBTQ discourse and bill. However, it quickly became a significant external threat to the successful implementation of the study. As noted in several discussions of policy and its impact on intervention implementation, national-level policies can significantly promote or hinder the success of interventions targeted at improving health outcomes among highly stigmatized people [28,29,30,31]. Like other countries, the criminalization of homosexuality, and the national anti-LGBTQ discourse in Ghana affect the ability of MSM to join interventions due to the fear of stigma, violence, and criminalization. The hostile climate may affect the implementation of interventions regardless of the novelty and effectiveness of the intervention [31,32,33]. Although a few participants declined to enroll in the study, the peer-to-peer mentoring space has built a support system for MSM who have fears about accessing HIV care services and/or going about their day-to-day duties. MSM communicate with their peers, ask questions, and receive referrals to MSM-friendly facilities for testing, thus, creating an avenue for continuous engagement with care despite the current anti-gay climate propelled by the bill.

Overall, the preliminary report shows that sexual health communication between peers can promote a healthy lifestyle, HIV testing, and linkage to care. In their conversations, mentors have provided information on HIV, STI, and HIV testing, and referred participants to facilities for HIV care. Mentees who concealed their HIV status or health care concerns at the initial stages of the study gradually opened to their peer mentors. Specifically, mentees asked for referrals to treat STIs, such as genital warts. Some disclosed living with HIV, and asked for support on understanding nutrition, HIV, and available care in Ghana. These preliminary observations complement several studies that show that peer-led interventions, where peers provide support and referral to HIV services, significantly increased the odds of HIV testing, especially among MSM [14,34,35,36,37,38,39,40,41].

Working in a highly stigmatized environment can be unpredictable, hence, the need to build a trustworthy and sustainable recruitment strategy when working with key people, such as MSM. Consistent with our previous experience, working with community-based organizations enabled us to successfully recruit and implement the study irrespective of the political climate [1,9,13]. Although we recorded some decline in participation, we essentially have been successful because we work with MSM organizations that have built trust over the years with the MSM in their respective communities. They remain a source of information, support, and linkage to care for MSM. Our success in working with community-based organizations (CBOs) reechoes the assertion that CBOs can recruit and retain participants in interventions due to their already established and trusted relationship with the target population [42,43,44,45].

Finally, leveraging technology can provide additional security and easy accessibility to mentors, and facilitate acceptability and accessible communication and connection to information and services. Our initial success supports previous findings that established the acceptability and efficacy of mobile-based interventions in improving health communication, and increasing access to HIV testing among key populations, such as men who have sex with men [14,46,47,48]. In this study, using user-friendly mediums, such as WhatsApp, accessible to most smartphones, help in keeping participants connected. Replacing in-person with online interaction enabled us to enforce the security and anonymity of mentees easily. Participants can hide their identity and seek help without disclosing their identity or identifying the mentor. It stands essential to note that despite the successes achieved so far, using mediums like WhatsApp or texting technologies may pose some risk to privacy [48]. A participant reported misplacing his phone. In such an instance, a third-party person can see the content of the conversation if they find the phone. The same may be applied to those who share accommodation with other people. However, we remedied such concerns by ensuring that participants understood not to associate the conversation with their real identities, and to use WhatsApp security features, such as facial recognition and passcode, when opening conversation each time. As such, we have not received complaints of confidentiality breaches thus far.

## 5. Conclusions

We used the Dennis Peer Support Model to design a communication-based peer support intervention (HIVE^3^). We leveraged existing relationships between community partners and key populations, and recent technology to increase sexual health communication between peers in Ghana. Although we reported some preliminary findings, this paper focuses on describing the intervention without a conclusive outcome of the study. We will evaluate and publish the full results on the efficacy of the intervention when the study completes. However, progress so far indicates success in implementing peer support on mobile technologies and addressing the health needs of people within stigmatized environments. We strongly recommend that scholars and program implementers targeting key populations in stigmatized settings use community-based organizations and peer-to-peer communication approaches, as they have the potential to improve health outcomes due to the trust, security, and privacy they accord participants. The peer-to-peer understanding of their unique challenges in such environments enables HIV key populations to relate and employ effective communication techniques to support self-efficacy development, and increased health and wellbeing.

## Figures and Tables

**Table 1:** Description of module sessions and module exercises.

Session	Modules/Exercise Description
ONE: Build Rapport and Confidentiality between Peer Mentors, Mentees, and other Team Members. Create space for familiarization and setting of a conducive environment for the remainder of the sessions.	A. We created introductory activities, and used energizers, such as songs and body movements, to welcome mentors, and create conversation. B. Setting of ground rules for the training. Mentors brainstormed and created a list of rules and expectations on a flip chart; we discussed the list, and signed them to show adherence. Some key rules include punctuality, no disturbances, unnecessary noise, cell phones, respect for each other, etc. C. Expectations and objectives. Mentors created a list of expectations, and we provided an oversight on the drivers of the intervention. D. Characteristics of a peer mentor. We use the same process as B and C to allow mentors to establish their understanding of a peer mentor, and their best characteristics. We then discussed the characteristics, and how to exhibit such characteristics. E. Confidentiality exercise. We created an activity to show the importance of confidentiality, and discussed the role of confidentiality in peer mentoring.
TWO: Understand Intersectional Stigma and How it Impacts HIV Care for MSM. Build participant understanding of the connection between stigma and health seeking behaviors/experiences (e.g., HIV testing, and retention to care), and ways to reduce intersectional stigma.	A. Stigma, Discrimination, and Intersectionality. We led a discussion on understanding stigma by asking mentors to define stigma, and provide examples supporting their explanations with a working definition. We then used examples in the Ghanaian context to depict intersectional stigma. We then implemented a matching terminology activity where pairs try to identify corresponding definitions related to our intervention; examples include stigma, outing, closeted, etc. B. MSM Experiences with Intersectional Stigma. Mentors reflected, shared, and discussed their personal experiences living as MSM in Ghana. C. HIV and Stigma. Mentors discussed in groups the meaning of HIV-related stigma, why HIV stigma exists, and how it affects people living with HIV, and discussed the consequences of stigma within healthcare sectors in their experiences. D. Reducing Stigma: Mentors brainstorm on reducing intersectional stigma in Ghana, and shared their personal recommendations through group discussions.
THREE: The Dennis Peer Support Model and the Role of Peer Mentors. Create peer mentors’ understanding on the DPSM, and its components on peer support (emotional, informational, and appraisal), and examine the role of peer mentors in providing support on dealing with intersectional stigma.	A. DPSM and its components. We used a PowerPoint presentation to explain the DPSM model and the various components. We then split mentors into groups to discuss the various components: emotional; information; appraisal/affirmatory support; and the sources of such support for them as mentors. B. Emotional. We used focused group discussions to brainstorm personal difficulties or self-esteem threats facing MSM, gender non-conforming MSM, and HIV+ MSM in Ghana (i.e., doubts about ability, social attractiveness), and established some of the mechanisms of emotional support (i.e., expressions of caring, encouragement, attentive listening, reflection, reassurance, and commonly avoiding criticism or exhortatory advice-giving) that they could offer mentees. C. Informational. Mentors defined information support and situations where they could provide informational support (HIV symptoms, questions about medication usage or side-effects, questions about other support groups for MSM or PLWHA in Ghana). D. Appraisal/Affirmational. Mentors examined affirmational support and the mechanisms of affirmational support (i.e., encouragement to persist in problem resolution, reassurances that efforts will result in positive outcomes, assistance to endure frustration, and communication of optimism). E. Distinguish Peer Support. We read scenarios, and asked mentors to explain the kind of support needed, and we provided additional explanations when needed. Examples of scenarios (e.g., an individual wants to disclose their HIV status to their parents, but is nervous, and he asks if he can walk through the conversation with you; an individual has a partner who is concerned they might have been infected with HIV; they want to know the nearest free testing center). F. Identifying Peer Support Need. Mentors formed groups, and created a play of their choice showing an MSM experiencing at least two intersecting stigmas at a health care facility, and they acted it out to allow the other peers to identify the kind of support the person needed.
FOUR: Effective Communication in Delivering Peer Support. Implementing emotional, and appraisal/affirmatory support using strategies for effective communications, empathetic listening and texting, as well as the strategy of LARA.	A. Effective Communication Strategies. Mentors discussed communication and its importance in groups, and facilitators provided a working definition and strategies (such as, focus on the issue and not the person, be genuine and not manipulative, show empathy, be flexible and open-minded, share experiences, ask questions, and express positive feelings, the 5Cs of effective communication—clear, cohesive, complete, concise, and concrete). B. Empathetic Listening and Texting. Mentors discussed empathetic listening and texting (paying attention, showing empathy, emotional identification, compassion, feeling, insight, the basic principle “seek to understand, before being understood”). They practice listening in role plays, and texting via WhatsApp. C. Non-Violent Communication: LARA. We provided an overview of LARA: listen (very carefully); affirm a feeling or value you share with the client; respond directly to the concerns or questions the client has raised; ask questions or add information. Mentors then practiced using their scenarios.
FIVE: Self-Efficacy. Develop self-efficacy and effective communication on sexual health, HIV, and risk reduction strategies among MSM.	A. Self-Efficacy. The purpose of this module is to enable mentors understand self-efficacy and ways to develop self-efficacy. We provided an overview of self-efficacy, and reviewed the different ways self-efficacy can be developed with mentors (e.g., performance accomplishment, verbal persuasion, physiological states). We discussed ways of withdrawing from difficult tasks, lack of concentration, energy spent focusing on limitations and failures, etc. C. Negative/Positive Message Exercise. Mentors discussed how self-talk affects self-efficacy. They created statements on why they believe they cannot be successful as MSM/as a PLWHA (“I don’t think I am a model for living with HIV/AIDS”, “I don’t think I am motivated enough to live with my illness.”, or “I am too effeminate to be considered a man”, etc.), and discarded the statements, and rewrote positive versions of the statements to apply in their lives. D. Motivational Interviewing. We discussed how mentors can develop interview skills which will foster social support (asking permission, eliciting/evoking change, provoking extremes, looking forward, importance and confidence ratings, open-ended questions). They practiced interviewing using these tips for feedback. E. Resources for self-care and effective coping. Mentors discussed the difference between effective coping and self-efficacy (i.e., focus on action instead of belief of self-worth or ability to accomplish given task; self-care—activities and practices that we can engage in on a regular basis to reduce stress, and maintain and enhance our short- and longer-term health and wellbeing).
SIX: Other Areas of Support, Usage of HIVE^3^ Platform, Signing up Gain the knowledge and skills to deal with other issues, such as economic stress and other types of stress that MSM go through. Train peer mentors to use the mobile application to link peer groups from the HIVE^3^ platform to operate as sub-groups.	A. Recap from the previous session. Mentors shared lessons from the previous sessions, and asked questions for clarifications. B. Dealing with economic stress and other stress. We provided an overview of economic related stress, family stress, psychosocial stress, etc., and how to deal or manage these stresses. We examine ways to deal with economic stress, for example, how to be entrepreneurial. C. Training on the Use of Mobile Application. We discussed the use of mobile phones and applications to manage discussion with peer mentors. Phones were given t o mentors on a later date, and they currently use them to provide support to participants.

## Data Availability

Not applicable.
